# Vertically Aligned Ultrathin 1T-WS_2_ Nanosheets Enhanced the Electrocatalytic Hydrogen Evolution

**DOI:** 10.1186/s11671-018-2570-x

**Published:** 2018-05-31

**Authors:** Qunying He, Longlu Wang, Kai Yin, Shenglian Luo

**Affiliations:** 1 State Key Laboratory of Chemo/Biosensing and Chemometrics, Hunan University, Changsha, 410082 People’s Republic of China; 2 School of Physics and Electronics, Hunan University, Changsha, 410082 People’s Republic of China

**Keywords:** Electrocatalysis, Hydrogen evolution reaction, WS_2_ nanosheets, Metallic 1T phase

## Abstract

**Abstract:**

Efficient evolution of hydrogen through electrocatalysis holds tremendous promise for clean energy. The catalytic efficiency for hydrogen evolution reaction (HER) strongly depends on the number and activity of active sites. To this end, making vertically aligned, ultrathin, and along with rich metallic phase WS_2_ nanosheets is effective to maximally unearth the catalytic performance of WS_2_ nanosheets. Metallic 1T polymorph combined with vertically aligned ultrathin WS_2_ nanosheets on flat substrate is successfully prepared via one-step simple hydrothermal reaction. The nearly vertical orientation of WS_2_ nanosheets enables the active sites of surface edge and basal planes to be maximally exposed. Here, we report vertical 1T-WS_2_ nanosheets as efficient catalysts for hydrogen evolution with low overpotential of 118 mV at 10 mA cm^−2^ and a Tafel slope of 43 mV dec^−1^. In addition, the prepared WS_2_ nanosheets exhibit extremely high stability in acidic solution as the HER catalytic activity and show no degradation after 5000 continuous potential cycles. Our results indicate that vertical 1T-WS_2_ nanosheets are attractive alternative to the precious platinum benchmark catalyst and rival MoS_2_ materials that have recently been heavily scrutinized for hydrogen evolution.

**Graphical Abstract:**

Vertical 1T-WS2 for hydrogen evolution.
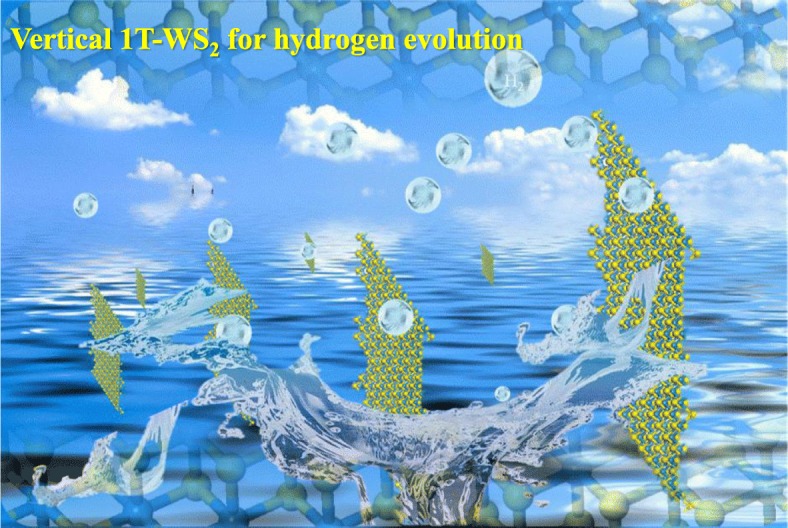

**Electronic supplementary material:**

The online version of this article (10.1186/s11671-018-2570-x) contains supplementary material, which is available to authorized users.

## Background

Hydrogen, as a clean fuel, has been considered as a promising alternative for traditional fossil fuels in the future [1, 2]. A tremendous amount of effort thus has been made to pursue sustainable and efficient hydrogen production. The electrocatalytic hydrogen evolution reaction (HER) is considered one of the most important pathways to produce hydrogen efficiently [3–5]. The most effective HER electrocatalysts up to now are based noble metals (e.g., platinum and palladium) [6, 7]. However, the high cost and scarcity of noble metals largely impede their practical utilization. Therefore, developing effective HER electrocatalysts with cheap and earth abundance still remains urgent.

In the search for nonprecious metal catalysts for the HER, transition metal dichalcogenides (TMDCs) have been proposed as promising candidates [8–21]. WS_2_-based electrocatalysts have been extensively investigated due to their high abundance and cost-efficiency [22–27]. However, bulk WS_2_ is a poor HER catalyst. At present, the effective routs for the synthesis of monolayer or few layers TMDCs nanosheets are chemical exfoliation and chemical vapor deposition (CVD). Normally, the chemical exfoliation needs n-butyllithium, which is a dangerous solvent resulting from the highly pyrophoric property in air [28–31]. CVD method incurs expensive apparatus, high temperature, and vacuum [32–34]. Therefore, an effective and environment-friendly strategy for large-scale preparation of ultrathin WS_2_ nanosheets is highly desirable.

Both experimental and computational studies confirm that the HER activity of TMDCs was mainly resulting from the rare edge surfaces, rather than basal planes [35, 36]. Stimulated by this understanding, intense investigations have been concentrated on developing highly nanostructured TMDCs to maximize the number of exposed edge sites, including crystalline and amorphous materials [37–41], metallic 1T polymorph [42, 43], vertically aligned structures [44, 45], and molecular mimics [46]. Although outstanding accomplishment, many actual challenges yet need to enhance the activity and stability of WS_2_-based catalysts.

Herein, we highlight a pathway to fulfill the assignment. Ultrathin WS_2_ nanosheets with perpendicular orientation and 1T metallic phase feature exhibit high activity and stability towards HER in acidic water. Its fast kinetic metrics (e.g., the Tafel slope of 43 mV dec^−1^) indicate superior electrocatalytic activity. This study hints at the promise of cheap and efficient HER electrocatalysts by one-step hydrothermal process.

## Experimental Section

### Synthesis of the Vertical 1T-WS_2_ Nanosheets

Vertical 1T-WS_2_ nanosheets were manufactured by a simple hydrothermal method on titanium substrate. In a typical procedure, thiourea (CS(NH_2_)_2_, i.e., 0.4104 g) and hexaammonium heptatungstate ((NH_4_)_6_W_7_O_24_, i.e., 0.267 g) were dissolved in 32 mL deionized water under vigorous stirring to form a homogeneous solution. Titanium substrate (1 × 4 cm) was carefully cleaned with concentrated hydrochloric solution, deionized water, and absolute ethanol in an ultrasound bath each for 10 min. The titanium substrate (against the wall) and the aqueous solution were transferred to a 40 mL Teflon-lined stainless steel autoclave. The autoclave was sealed and maintained at 200 °C for 7 h and then enabled to cool down to room temperature within 15 min using cooling water. A dark thin film was extracted from the autoclave and subsequently rinsed with deionized water and absolute ethanol, and dried at 60 °C under vacuum. The loading mass of WS_2_ nanosheets was determined by weighing the titanium substrate before and after hydrothermal process; a surface density of approximately 100 μg cm^−2^ was obtained.

### Synthesis of the Flat 1T-WS_2_ Nanosheets

For the synthesis of flat 1T-WS_2_ nanosheets, 0.267 g (NH_4_)_6_W_7_O_24_ and 0.4104 g CS(NH_2_)_2_ were dissolved in 32 mL deionized water under vigorous stirring to form a clear solution. Then, the solution was transferred into a 40 mL Teflon-lined stainless steel autoclave, maintained at 200 °C for 7 h, and allowed to cool to room temperature naturally. The final product was washed with deionized water and absolute ethanol for several times and dried at 60 °C under vacuum. Specifically, the obtained WS_2_ catalyst was dispersed in an ethanol solution with a concentration of 0.8 mg ml^−1^. Then, we loaded the WS_2_ catalyst or Pt/C on titanium substrate by a drop-casting method with a mass loading of approximately 100 μg cm^−2^ as well. All the materials were purchased from SinoPharm and used without further purification.

### Characterization

The morphologies and microstructures of WS_2_ nanosheets were characterized via field emission scanning electron microscope (FESEM, Hitachi, Japan) and transmission electron microscopy (TEM, Tecnai F20). The energy-dispersive X-ray spectroscopy (EDS) mapping images were captured on a Tecnai G2 F20 S-TWIN atomic resolution analytic microscope. The binding energies of W and S were determined by X-ray photoelectron spectroscopy (XPS, K-Alpha 1063, Thermo Fisher Scientific, England) using an Al-Kα X-ray source.

### Electrochemical Measurements

All electrochemical measurements were performed at room temperature on a standard three-electrode electrolytic system. The saturated calomel electrode (SCE), carbon stick electrode and titanium substrate growth directly with WS_2_ nanosheets were served as reference electrode, counter and working electrode, respectively. As for reference, titanium substrate with deposited Pt/C and WS_2_ nanosheets (approximately 100 μg cm^−2^) also was regarded as working electrode. The HER activities were conducted by linear sweep voltammetry (LSV) solution with a scan rate of 5 mV s^−1^. The stability was tested by taking continuous cyclic voltammograms at a scan rate of 50 mV s^−1^ from − 0.4 to 0.1 V with 5000 cycles. The striking stability was further demonstrated by using chronoamperometry (j~t) at 160 mV. All the measurements were performed in 0.5 M H_2_SO_4_ without *i*R compensated. The electrolyte solution was purged with high purity nitrogen (N_2_) for half an hour to remove the dissolved oxygen before testing. Under without special emphasis, all the potentials were here referenced to the reversible hydrogen electrode (RHE) using the following equation:

$$ \mathrm{E}\left(\mathrm{RHE}\right)=\mathrm{E}\left(\mathrm{SCE}\right)+0.24\ \mathrm{V}+0.059\times \mathrm{pH} $$

## Results and Discussion

### Characterization Supports of Catalysts

**Fig. 1:**
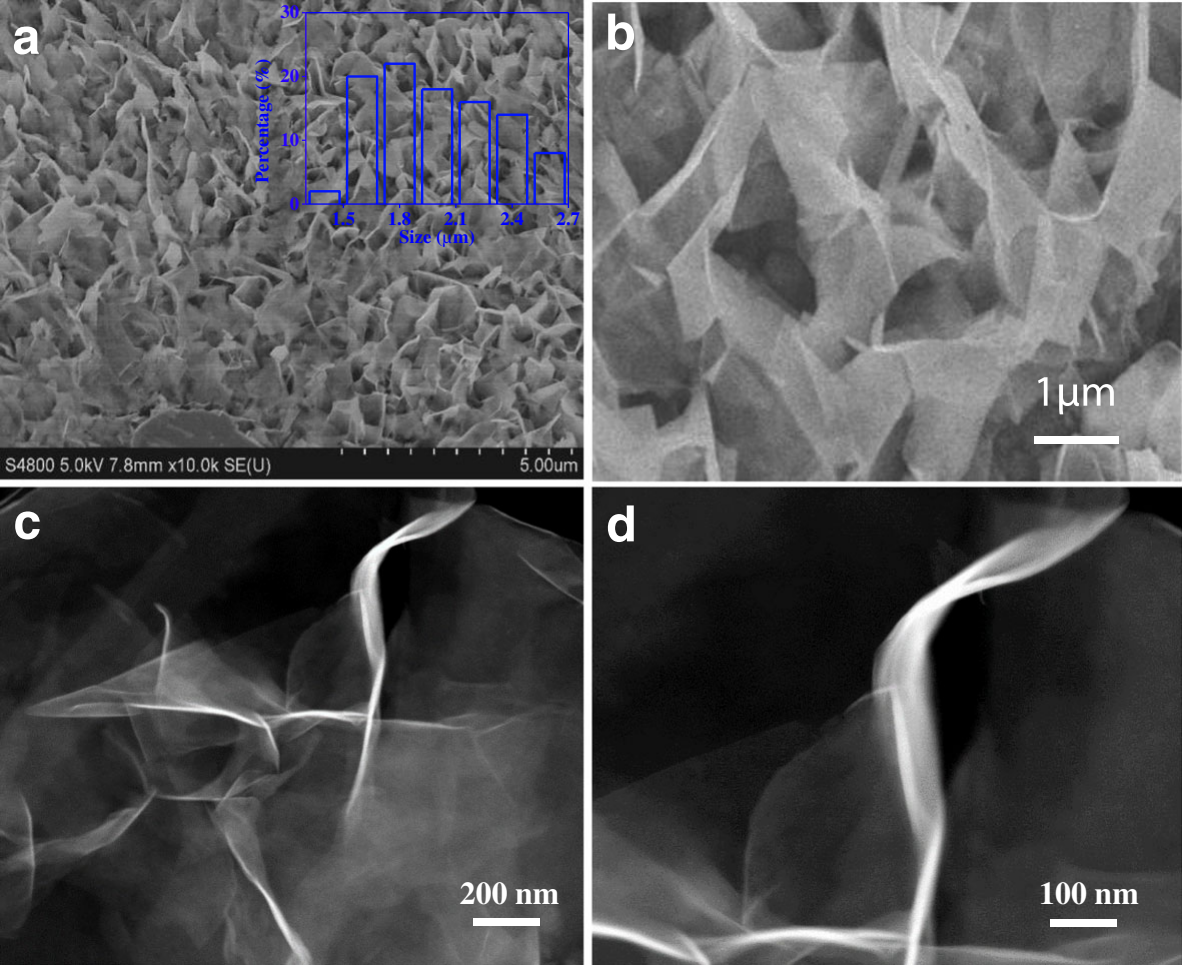
**a**–**b** Top-down SEM image of the prepared vertical 1T-WS_2_ nanosheets on Ti substrate. **c**–**d** HAADF-STEM of 1T-WS_2_ nanosheets

Figure 1a shows the scanning electron microscopy (SEM) image of the prepared vertical 1T-WS_2_ nanosheets with dimensions of ca. 2 μm, which indicated that nanosheets were exceedingly large. As shown in Fig. 1b, the nanosheets are nearly perpendicular to the electrode Ti substrate, which facilitates the exposure of WS_2_ edge sites as edge-oriented grapheme on carbon nanofiber [47]. The cross profile of vertical 1T-WS_2_ nanosheets is shown in Additional file 1: Figure S1. Meanwhile, crisscross rather than stack occurred between nanosheets. Such an open structure is supposed to allow the fast transportation of proton throughout the catalyst and utilize the basal planet sites for HER as well. Vertical 1T-WS_2_ nanosheets in Fig. 1c are extremely transparent, implying that formed nanosheets were ultrathin. The noticeable distortion of nanosheets (Fig. 1d) helps to decrease their high surface energy to make the WS_2_ stable as independent ultrathin nanosheet units. Meanwhile, the luminous line in Fig. 1c, d indicated that prepared WS_2_ nanosheets hold excellent conductivity, which is vital for electrocatalytic HER.

**Fig. 2:**
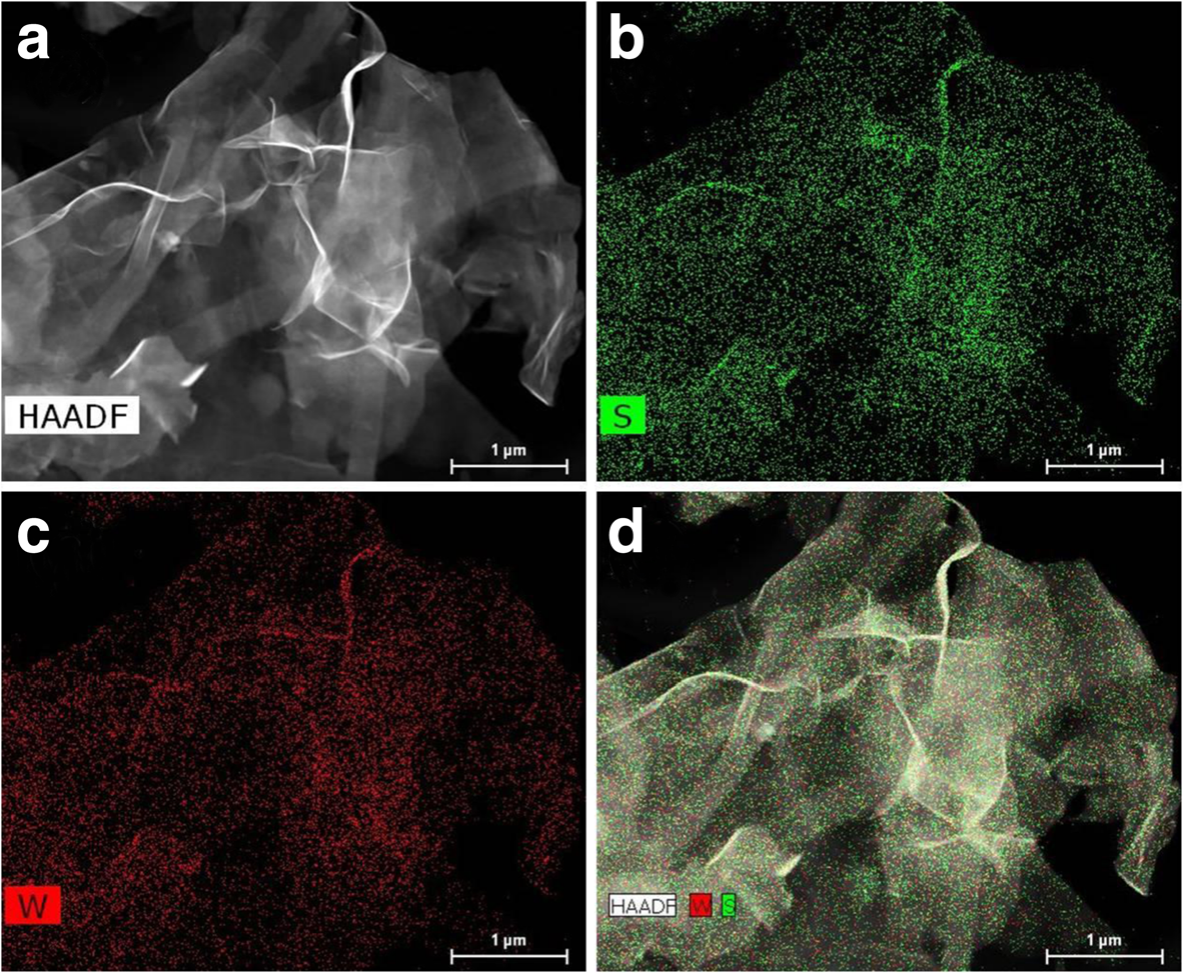
HAADF-STEM image (**a**) and corresponding elemental mapping (**b** for S, **c** for W, **d** for S and W) for the 1T-WS_2_ nanosheets

The HAADF-SEM image (Fig. 2a) and homogeneously distributed W and S component elements from the corresponding energy-dispersive X-ray (EDX) mapping (Fig. 2b, c) further reveal the successful synthesis of WS_2_ nanosheets. In addition, the elemental mapping overlapping of S and W (Fig. 2d) was dovetailing well and evidenced convincingly the WS_2_ nanosheets formed. Meanwhile, elemental analysis using EDS shows the homogeneous distribution of W and S in WS_2_ nanosheets (Additional file 1: Figure S2).

**Fig. 3:**
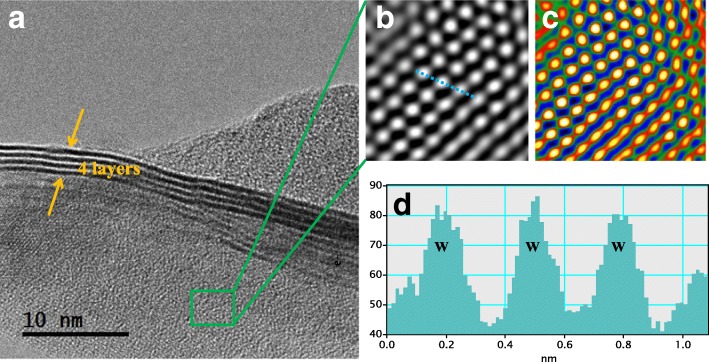
HRTEM image of **a** vertical 1T-WS_2_ nanosheets and **b**, **c** false-color images responding to the amplification of **a**. Intensity profiles along the light-blue line indicated in image **b** is shown in image **d**

The precise microscopic knowledge of nanostructure materials is of fundamental importance. In Fig. 3a, the high-resolution TEM image (HRTEM) shows the disordered structure of WS_2_ nanosheets. Moreover, these WS_2_ nanosheets with a thickness of about four layers are dominated by well-defined crystalline edges, thus increasing the density of active sites. To better understand the atomic structure, we have further utilized the Z-contrast. As shown in Fig. 3b, c, the crystal structure of the sheets is not the hexagonal packing usually observed for 2H-WS_2_ but rather corresponding to 1T-WS_2_ structure. It is obvious that S atoms are evenly distributed between the W and W sites to form a 1T phase, as shown in Fig. 3d. Meanwhile, metallic 1T phase could be converted into semiconducting 2H phase after 300 °C annealing treatment, as shown in Additional file 1: Figure S3.

**Fig. 4:**
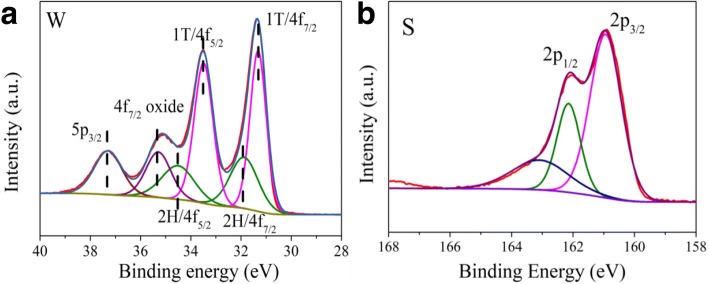
XPS spectra of W 4f (**a**) and S 2p (**b**) binding energy of vertical 1T-WS_2_ nanosheets

X-ray photoelectron spectroscopy (XPS) was able to confirm the chemical state and composition. All XPS spectra were calibrated using the C 1s peak at 284.8 eV. Meanwhile, XPS could distinguish 1T- and 2H-WS_2_ as well. As shown in Fig. 4a, the 2H-WS_2_ features two characteristic peaks at around 34.49 and 31.94 eV, corresponding to W4f_5/2_ and W4f_7/2_ of 2H-WS_2_ components, respectively, while the 1T-WS_2_ displays the presence of new chemical species clearly shifted toward lower binging energies (33.54 and 31.29 eV, corresponding to W4f_5/2_ and W4f_7/2_ of 1T-WS_2_ components) [48]. The result suggests nanosheets were the mixture of 1T- and 2H-WS_2_. The nanosheets also contain a small amount of tungstate, as evidenced by the signal at 35.14 eV, which corresponds to a W4f_7/2_ species. These results are consistent with the known metallic nature of 1T-WS_2_ nanosheets, which are susceptible to oxidation [28]. It is worth noting that a slight oxidation of TMDs can improve the density of the active sites, which can enhance the catalytic activities of nanosheets. Nonetheless, exhaustive oxidation should be avoided [10]. The relative percentages of 1T-WS_2_ and 2H-WS_2_ obtained by integration of the W4f_7/2_ peak were 70 and 30%, respectively. Such high concentration of the metallic phase in WS_2_ nanosheets may lead to a dramatic enhancement in the catalytic activities [30]. Such phase conformation was desired in electrocatalytic hydrogen evolution. Simultaneously, S 2p region of the spectra (Fig. 4b), the peaks located at 161.6 and 162.7 eV, are assigned to S2p_3/2_ and S2p_1/2_, respectively [49]. Moreover, the atom ratio of W and S in the vertical 1T-WS_2_ nanosheets by XPS and ICP (in Additional file 1: Table S1) was 1:1.96 and 1:1.94, respectively.

**Fig. 5:**
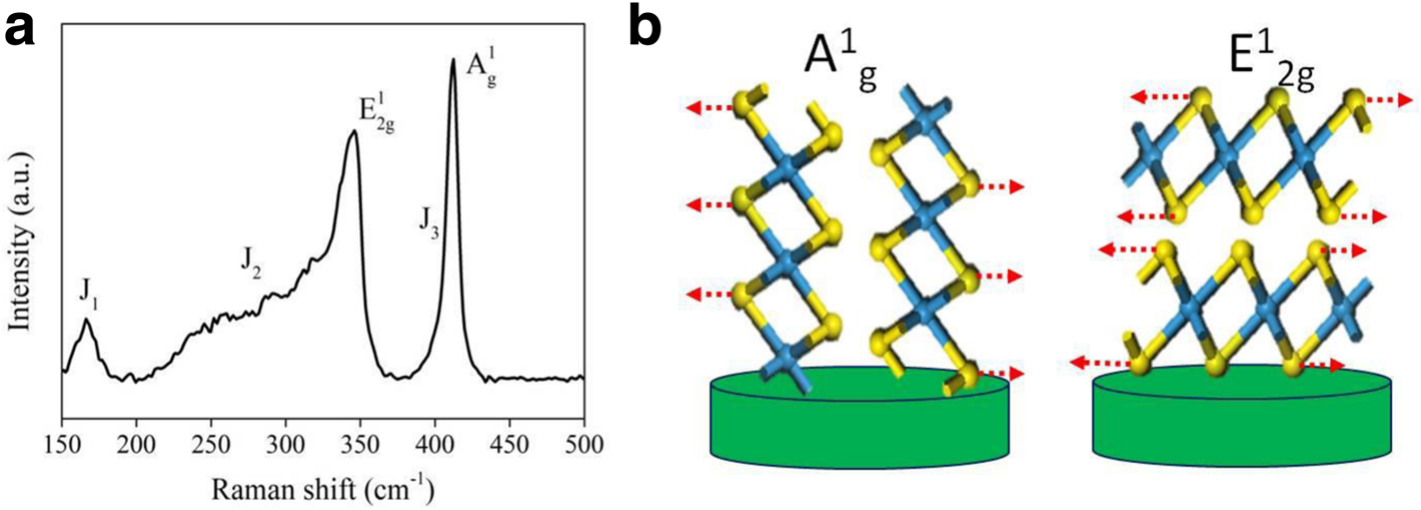
**a** Raman spectrum of vertical 1T-WS_2_ nanosheets. **b** Schematics of preferentially excited A^1^_g_ Raman mode for edge-terminated nanosheets (top) and E^1^_2g_ mode for terrace-terminated nanosheets (bottom)

Raman spectroscopy measurements were also performed to further confirm the phase classification. Figure 5a presents Raman spectra collected from vertical 1T-WS_2_ nanosheets grown on Ti substrate. Due to the polarization dependence, out-of-plane A^1^_g_ is preferentially excited for edge-terminated nanosheets, whereas the in-plane E^1^_2g_ is preferentially excited for terrace-terminated nanosheets, as illustrated in Fig. 5b. The characteristic Raman shifts at 343 and 411 cm^−1^ expected for the E^1^_2g_ and A^1^_g_ were clearly observed, respectively [50]. In addition, the additional peaks in the lower frequency regions were previously referred as J1, J2, and J3, corresponding to modes that were only in 1T-type WS_2_ and not allowed in 2H-WS_2_ [22]. In the Additional file 1: Figure S4, the J1, J2, and J3 peaks after annealing were quenched, which also verify the transformation from 1T phase to 2H phase. These interpretations together with the aforementioned characterization results solidly confirm the formation of vertical 1T-WS_2_ nanosheets.

### Evaluation of Electrocatalytic Activity

To assess electrocatalytic performance of vertical 1T-WS_2_ nanosheets in HER, measurements are performed in a 0.5 M H_2_SO_4_ solution using a typical three-electrode cell setup. For reference purposes, Ti substrate with a drop-cast commercial Pt benchmark (Pt/C) and WS_2_ nanosheets catalysts has also been used as the working electrode.

**Fig. 6:**
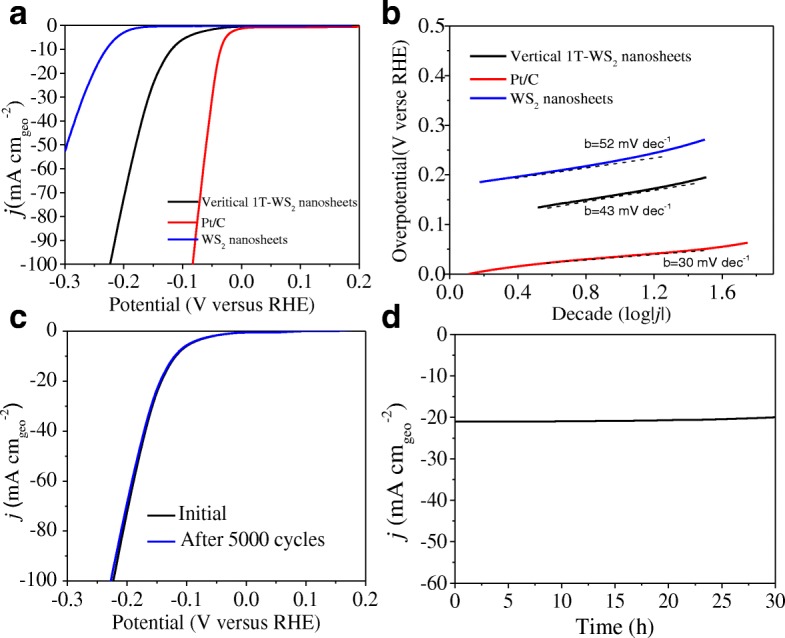
**a** Polarization curves and **b** Tafel plots of Pt/C, WS_2_ nanosheets, and vertical 1T-WS_2_ nanosheets in 0.5 M H_2_SO_4_ at a scan rate of 5 mV/s. **c** Durability test showing negligible current loss even after 5000 CV cycles and **d** time dependence of the current density curve at an overpotential of 160 mV versus RHE for vertical 1T-WS_2_ nanosheets (no *i*R compensation)

The polarization curves of all samples are shown in Fig. 6a. The vertical 1T-WS_2_ nanosheets exhibit a low overpotential of 118 mV (V vs RHE), compared to the overpotential of 230 mV for WS_2_ nanosheets at 10 mA cm^−2^. It indicated that rich metallic polymorph (~ 70%) in basal planes and exposed edge sites of vertical 1T-WS_2_ nanosheets can significantly increase the electrochemical HER activity. In addition, the structure of vertical 1T-WS_2_ nanosheets guarantees efficient charge flow from the conductive support to active surface site along individual layers. It is in fact a general consideration in designing TMDCs HER catalysts to minimizing ohmic loss, as the interlayer conductivity is 2 order of magnitude lower than intralayer conductivity [8, 51]. Electrons are required to traverse the van der Waals gaps to move between the individual layers; therefore, vertical nanostructure does favor for electrons shuttle [44]. Besides, the vertical 1T-WS_2_ nanosheets after annealing at 300 °C were investigated as well (in Additional file 1: Figure S5), and the hydrogen evolution performance significantly decrease.

Tafel plot in Fig. 6b is used to determine the Tafel slope, which is an important parameter describing HER activity of catalysts. The linear part of vertical 1T-WS_2_ nanosheets Tafel plot under small overpotential is fitted to give a Tafel slope of 43 mV dec^−1^, which is smaller than those of previously reported values (in Table 1 and Additional file 1: Table S2, including WS_2_/MoS_2_-based catalysts). Tafel slope is associated with the elementary steps in HER. The first step of HER is a discharge step (Volmer reaction, Eq. 1) in which protons are adsorbed to active sites on the surface of the catalysts and combined with electrons to form adsorbed hydrogen atoms. It is followed by a desorption step (Heyrovsky reaction, Eq. 2) or a combination step (Tafel reaction, Eq. 3) [52, 53].

1 $$ {\mathrm{H}}_3{\mathrm{O}}^{+}+{\mathrm{e}}^{-}\to {\mathrm{H}}_{\mathrm{ads}}+{\mathrm{H}}_2\mathrm{O} $$

2 $$ {\mathrm{H}}_{\mathrm{ads}}+{\mathrm{H}}_3{\mathrm{O}}^{+}+{\mathrm{e}}^{-}\to {\mathrm{H}}_2+{\mathrm{H}}_2\mathrm{O} $$

3 $$ {\mathrm{H}}_{\mathrm{ads}}+{\mathrm{H}}_{\mathrm{ads}}\to {\mathrm{H}}_2 $$

**Table 1:** Summary of literature catalytic parameters of various WS_2_ or WS_2_-based catalysts, recently

Catalysts	Onset overpotential [mV]	Tafel slopes [mV decade^−1^]	η@ j = 10 mA cm^−2^ [mV]	Ref.
1T-WS_2_ nanosheets	~ 100	60	250	[22]
WS_2_ nanoribbons	–	109	> 420	[24]
WS_2_ NRs-CH_3_OH	–	86	260	
WS_2_ NRs-H_2_O	–	68	225	
WS_2_ NRs-250 °C	–	97	313	
2H-WS_2_ nanoflake	100	48	–	[25]
WS_2_ NDs	90	51		[26]
WS_2_ NDs − 300 °C	180	59		
Bulk-WS_2_	270	119		
2H-WS_2_ nanosheets	60	72	~ 160	[54]
2H-WS_2_	282	110	–	[55]
Au/2H-WS_2_	233	57.5	–	
Annealed WS_2_		140	–	[56]
WS_2_/rGO	150–200	58	–	
WS_2_ nanotubes	–	113	–	[57]
VA WS_2_ nanosheets	30	61	136	[58]
WS_2_ nanosheets	–	97	236	[59]
rGO/WS_2_ nanosheets	–	73	229	
1T-WS_2_ nanosheets	100	43	118	This work

Under a special set of conditions, when the Volmer reaction is the rate-determining step of HER, a slop of ca. 120 mV dec^−1^ should result, while a rate-determining Heyrovsky of Tafel reaction should produce slope of ca. 30 and 40 mV dec^−1^, respectively [52, 53]. In this work, it seems that free energy barrier of discharge step is reduced to be comparable with that of the following desorption or combination step, resulting in the slope of 43 mV dec^−1^ for vertical 1T-WS_2_ nanosheets. Meanwhile, the key step in HER is the adsorption of the proton on the active site. To asses this, we have varied the pH, as shown in Additional file 1: Figure S6. We found that the vertical 1T-WS_2_ nanosheets are active over a wide range of pH although the activity decreases when increasing the pH from 0 to 7, which results from the strong diminution of the quantity of protons available.

Stability is another important criterion for electrocatalysts. To assess the long-term durability of vertical 1T-WS_2_ nanosheets in an acid environment, continuous HER by CV in the cathodic potential window at an accelerated scanning rate of 5 mV/s were conducted. The polarization curves before and after cycling are recorded under quasi-equilibrium conditions. Polarization curves after the 5000 cycles almost overlay the curve of the initial cycle with negligible loss of cathodic current, as shown in Fig. 6c. It confirms that vertical 1T-WS_2_ nanosheets are stable in acidic electrolyte and remain intact through repeated cycling. Meanwhile, vertical 1T-WS_2_ nanosheets associated ability to continuously catalyze the generation of H_2_ was examined using chronoamperometry (*j*-t). This quasi-electrolysis process was conducted at a constant of 160 mV in 0.5 M H_2_SO_4_ (Fig. 6d). Remarkably, the H_2_ evolution can proceed at a sustained current density of − 21 mA cm^−2^ even over 30 h of continuous operation, indicating the ultrahigh stability of vertical 1T-WS_2_ nanosheets.

## Conclusions

In summary, we have developed a simple, eco-friendly, and effective hydrothermal method for the synthesis of vertical 1T-WS_2_ nanosheets. The vertical 1T-WS_2_ nanosheets, with metallic polymorph and exposed edge sites, represent a novel structure of layered materials. The unique structure paves the ways to utilize the edges and planes of layered materials more effectively. Hence, such nanostructure catalysts combined with the scalability of the hydrothermal synthesis can be readily applied in diverse water electrolysis as low-cost, high-performance, and stable HER catalyst.

## Additional file

Additional file 1: **Fig S1.** The cross profile SEM image of the prepared vertical 1T-WS_2_ nanosheets on Ti substrate. **Fig S2.** Whole-energy spectra of vertical 1T-WS_2_ nanosheets.** Fig S3.** (a) and (b) are false-color images responding to vertical 1T-WS_2_ nanosheets transform into 2H-WS_2_ nanosheets after 300 °C annealing treatment, respectively. **Fig S4.** Raman spectrum of vertical 1T-WS_2_ nanosheets (bottom) transform into 2H-WS_2_ nanosheets (up) after 300 °C annealing treatment. **Fig S5.** Polarization curves of vertical 1T-WS_2_ nanosheets after annealing at 300 °C in 0.5 M H_2_SO_4_ at a scan rate of 5 mV/s. **Fig S6.** Variation of current density versus the potential as a function of the pH for the vertical 1T-WS_2_ nanosheets. The highest current density is obtained for the lowest pH, consistent with the solution having the highest proton concentration. **Table S1.** Element analyses of the vertical 1T-WS_2_ nanosheets. **Table S2.** Summary of literature catalytic parameters of various MoS_2_ or MoS_2_-based catalysts, recently. (DOCX 1570 kb).

## Abbreviations

CVD: Chemical vapor deposition
EDS: Energy-dispersive X-ray spectroscopy
HER: Hydrogen evolution reaction
LSV: Linear sweep voltammetry
RHE: Eversible hydrogen electrode
SCE: Saturated calomel electrode
TEM: Transmission electron microscopy
TMDCs: Transition metal dichalcogenides
XPS: X-ray photoelectron spectroscopy
