# Supplementary material for: Vertically Aligned Ultrathin 1T-WS2 Nanosheets Enhanced the Electrocatalytic Hydrogen Evolution
Source: Nanoscale Res Lett. 2018 May 31;13:167. doi: 10.1186/s11671-018-2570-x (PMC5981154; doi:10.1186/s11671-018-2570-x)
Supplement: Supplementary file 1 — Fig S1. The cross profile SEM image of the prepared vertical 1T-WS2 nanosheets on Ti substrate. Fig S2. Whole-energy spectra of vertical 1T-WS2 nanosheets. Fig S3. (a) and (b) are false-color images responding to vertical 1T-WS2 nanosheets transform into 2H-WS2 nanosheets after 300 °C annealing treatment, respectively. Fig S4. Raman spectrum of vertical 1T-WS2 nanosheets (bottom) transform into 2H-WS2 nanosheets (up) after 300 °C annealing treatment. Fig S5. Polarization curves of vertical 1T-WS2 nanosheets after annealing at 300 °C in 0.5 M H2SO4 at a scan rate of 5 mV/s. Fig S6. Variation of current density versus the potential as a function of the pH for the vertical 1T-WS2 nanosheets. The highest current density is obtained for the lowest pH, consistent with the solution having the highest proton concentration. Table S1. Element analyses of the vertical 1T-WS2 nanosheets. Table S2. Summary of literature catalytic parameters of various MoS2 or MoS2-based catalysts, recently. (DOCX 1570 kb). [file 11671_2018_2570_MOESM1_ESM.docx]

**Supplementary Information for**

**Vertically Aligned Ultrathin 1T-WS_2_ Nanosheets Enhanced the Electrocatalytic Hydrogen Evolution**

Qunying He^1^, Longlu Wang^1,2*^, Kai Yin^1*^, Shenglin Luo^1^

^1^ State Key Laboratory of Chemo/Biosensing and Chemometrics, Hunan University, Changsha 410082, P. R. China.

^2^ School of Physics and Electronics, Hunan University, Changsha 410082, P. R. China

**Corresponding Author**

E-mail address: wanglonglu@hnu.edu.cn

E-mail address: [fantasyksky@hnu.edu.cn](mailto:fantasyksky@hnu.edu.cn)

Fig S1. The cross profile SEM image of the prepared vertical 1T-WS_2_ nanosheets on Ti substrate.


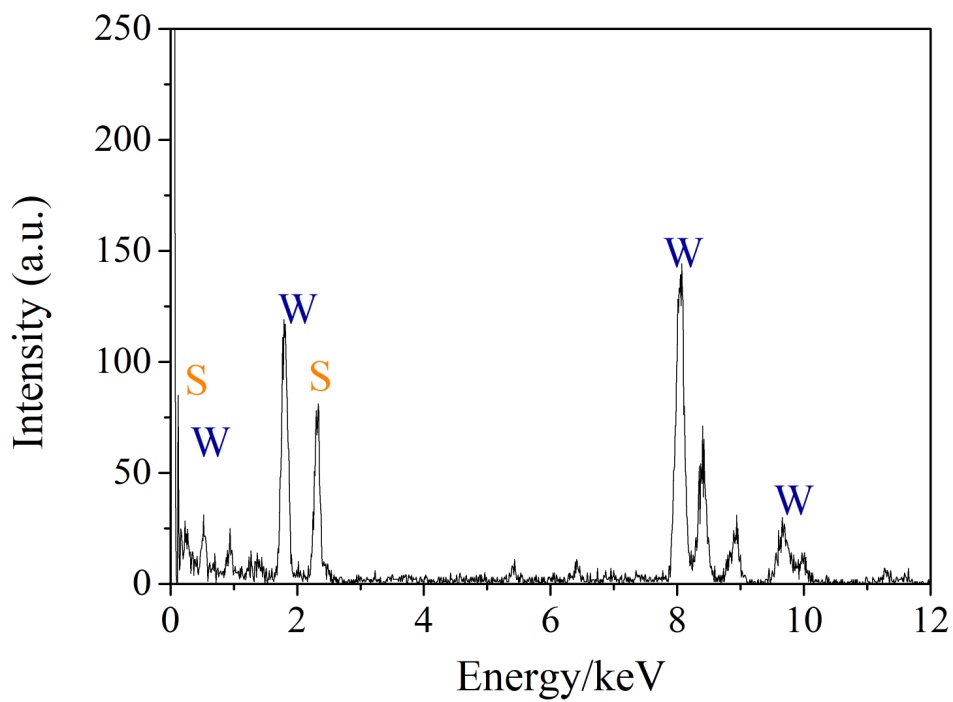


Fig S2. Whole-energy spectra of vertical 1T-WS_2_ nanosheets.

Fig S3. (a) and (b) are false-color images responding to vertical 1T-WS_2_ nanosheets transform into 2H-WS_2_ nanosheets after 300 ℃ annealing treatment, respectively.

Fig S4. Raman spectrum of vertical 1T-WS_2_ nanosheets (bottom) transform into 2H-WS_2_ nanosheets (up) after 300 ℃ annealing treatment.

Fig S5. Polarization curves of vertical 1T-WS_2_ nanosheets after annealing at 300 ℃ in 0.5 M H_2_SO_4_ at a scan rate of 5 mV/s.

Fig S6. Variation of current density versus the potential as a function of the pH for the vertical 1T-WS_2_ nanosheets. The highest current density is obtained for the lowest pH, consistent with the solution having the highest proton concentration.

Table S1. Element analyses of the vertical 1T-WS_2_ nanosheets.

| Sample | Atomic Ratio of W/S | |
| --- | --- | --- |
|  | By XPS | By ICP |
| Vertical 1T-WS_2_ | 1:1.96 | 1:1.94 |

Table S2. Summary of literature catalytic parameters of various MoS_2_ or MoS_2_-based catalysts, recently.

| Catalysts | Overpotential  [mV] | Tafel slopes  [mV decade^‐1^] | η@ j = 10 mA cm^-2^  [mV] | Ref. |
| --- | --- | --- | --- | --- |
| MoS_2_ nanosheets | 120 | 50 | ~190 | [1] |
| 1T-Verticallly MoS_2_ | 120 | 44 | ~180 | [2] |
| MoO_3_-MoS_2_ nanowires | 150-200 | 50-60 | ~250 | [3] |
| Dendritic MoS_2_ | 100-278 | 73-85 | - | [4] |
| MoS_2_ quantum dots | 190 | 74 | - | [5] |
| 1T MoS_2_ nanosheets | ~135 | 43 | ~187 | [6] |
| Amorphous MoS_2_ | 150 | 60 | 201 | [7] |
| Interlay-expanded MoS_2_ | 103 | 49 | 149 | [8] |
| Li-MoS_2_/carbon fiber | - | 62 | 118 | [9] |
| MoS_2_/CNT-grapheme | 255 | 43 | 100 | [10] |
| MoS_2_ hollow spheres | 112 | 74 | 214 | [11] |
| 1T@2H-MoS_2_ | 42 | 49 | 64 | [12] |
| CoS_2_/MoS_2_/CC | N/A | 66 | 177 | [13] |
| micro and ground microﬂakes -MoS_2_ | N/A | 60-70 | 174 | [14] |
| MoS_2_/Graphene | 30 | 67.4 | 110 | [15] |
| Hollow MoS_2_ | N/A | 48 | 202 | [16] |
| C/MoS_2_@G | 165 | 46 | N/A | [17] |
| Laser MoS_2_/carbon | N/A | 64 | 216 | [18] |
| DR‐MoS_2_ | 166 | 66 | 208 | [19] |

**References**

1. Xie J, Zhang H, Li S, Wang R, Sun X, Zhou M, Zhou J et al (2013) Defect‐rich MoS_2_ ultrathin nanosheets with additional active edge sites for enhanced electrocatalytic hydrogen evolution. Adv Mater, 25 (40): 5807-5813

2. Wang H, Lu Z, Xu S, Kong D, Cha J J, Zheng G, Hsu P-C et al (2013) Electrochemical tuning of vertically aligned MoS_2_ nanofilms and its application in improving hydrogen evolution reaction. Proceedings of the National Academy of Sciences, 110 (49): 19701-19706

3. Chen Z, Cummins D, Reinecke B N, Clark E, Sunkara M K, Jaramillo T F (2011) Core–shell MoO_3_–MoS_2_ nanowires for hydrogen evolution: a functional design for electrocatalytic materials. Nano Lett, 11 (10): 4168-4175

4. Zhang Y, Ji Q, Han G-F, Ju J, Shi J, Ma D, Sun J et al (2014) Dendritic, transferable, strictly monolayer MoS_2_ flakes synthesized on SrTiO_3_ single crystals for efficient electrocatalytic applications. ACS Nano, 8 (8): 8617-8624

5. Gopalakrishnan D, Damien D, Shaijumon M M (2014) MoS_2_ quantum dot-interspersed exfoliated MoS_2_ nanosheets. ACS Nano, 8 (5): 5297-5303

6. Lukowski M A, Daniel A S, Meng F, Forticaux A, Li L, Jin S (2013) Enhanced hydrogen evolution catalysis from chemically exfoliated metallic MoS_2_ nanosheets. J Am Chem Soc, 135 (28): 10274-10277

7. Benck J D, Chen Z, Kuritzky L Y, Forman A J, Jaramillo T F (2012) Amorphous molybdenum sulfide catalysts for electrochemical hydrogen production: insights into the origin of their catalytic activity. ACS Catal, 2 (9): 1916-1923

8. Gao M R, Chan M K, Sun Y (2015) Edge-terminated molybdenum disulfide with a 9.4-A interlayer spacing for electrochemical hydrogen production. Nat Commun, 6: 7493

9. Wang H, Lu Z, Kong D, Sun J, Hymel T M, Cui Y (2014) Electrochemical tuning of MoS_2_ nanoparticles on three-dimensional substrate for efficient hydrogen evolution. ACS Nano, 8 (5): 4940-4947

10. Youn D H, Han S, Kim J Y, Kim J Y, Park H, Sun H C, Lee J S (2014) Highly Active and Stable Hydrogen Evolution Electrocatalysts Based on Molybdenum Compounds on Carbon Nanotube–Graphene Hybrid Support. ACS Nano, 8 (5): 5164-73

11. Guo B, Ke Y, Li H, Song H, Zhang Y, Xiang L, Hao F et al (2016) Hollow Structured Micro/Nano MoS_2_ Spheres for High Electrocatalytic Activity Hydrogen Evolution Reaction. ACS Appl Mater Interfaces, 8 (8): 5517

12. Shi S, Gao D, Xia B, Liu P, Xue D (2015) Enhanced hydrogen evolution catalysis in MoS_2_ nanosheets by incorporation of a metal phase. J Mater Chem A, 3 (48): 24414-24421

13. Su C, Xiang J, Wen F, Song L, Mu C, Xu D, Hao C et al (2016) Microwave Synthesized Three-dimensional Hierarchical Nanostructure CoS_2_/MoS_2_ Growth on Carbon Fiber Cloth: A Bifunctional Electrode for Hydrogen Evolution Reaction and Supercapacitor. Electrochim Acta, 212: 941-949

14. Kiriya D, Lobaccaro P, Nyein H Y Y, Taheri P, Hettick M, Shiraki H, Sutter-Fella C M et al (2016) General thermal texturization process of MoS_2_ for efficient electrocatalytic hydrogen evolution reaction. Nano Lett, 16 (7): 4047-4053

15. Ma L, Hu Y, Zhu G, Chen R, Chen T, Lu H, Wang Y et al (2016) In Situ Thermal Synthesis of Inlaid Ultrathin MoS_2_/Graphene Nanosheets as Electrocatalysts for the Hydrogen Evolution Reaction. Chemistry of Materials, 28 (16): 5733-5742

16. Ambrosi A, Pumera M (2016) Templated electrochemical fabrication of hollow molybdenum sulfide microstructures and nanostructures with catalytic properties for hydrogen production. ACS Catal, 6 (6): 3985-3993

17. Li Y, Wang J, Tian X, Ma L, Dai C, Yang C, Zhou Z (2016) Carbon doped molybdenum disulfide nanosheets stabilized on graphene for the hydrogen evolution reaction with high electrocatalytic ability. Nanoscale, 8 (3): 1676-1683

18. Deng H, Zhang C, Xie Y, Tumlin T, Giri L, Karna S P, Lin J (2016) Laser induced MoS_2_/carbon hybrids for hydrogen evolution reaction catalysts. J Mater Chem A, 4 (18): 6824-6830

19. Qi K, Yu S, Wang Q, Zhang W, Fan J, Zheng W, Cui X (2016) Decoration of the inert basal plane of defect-rich MoS_2_ with Pd atoms for achieving Pt-similar HER activity. J Mater Chem A, 4 (11): 4025-4031
